# A Scoping Review of Camp Activities for Children with Developmental Disorders

**DOI:** 10.31662/jmaj.2025-0435

**Published:** 2026-03-19

**Authors:** Seiichiro Nagai, Tatsuki Ikejiri, Hayase Hakariya, Yuki Otsuka

**Affiliations:** 1 Medical Research Institute Kitano Hospital, Osaka, Japan; 2 Center for Clinical Education, Hikone Municipal Hospital, Hikone, Japan; 3 Interfaculty Institute of Biochemistry, University of Tuebingen, Tuebingen, Germany; 4 Department of General Medicine, Okayama University Graduate School of Medicine, Dentistry and Pharmaceutical Sciences, Okayama, Japan

**Keywords:** developmental disorders, children, camp activities, social model of disability

## Abstract

**Background:**

The growing number of children diagnosed with developmental disorders, such as autism spectrum disorder, attention deficit hyperactivity disorder, and learning disabilities, poses increasing challenges for social inclusion and support systems. Among various support interventions, nature-based camp activities have attracted attention for their potential to provide multifaceted benefits. This review aimed to clarify the value and effects of camp activities for children with developmental disorders, their families, and staff, based on contemporary perspectives that reflect changing views on disability and human-nature relationships.

**Methods:**

Following Preferred Reporting Items for Systematic reviews and Meta-Analyses extension for Scoping Reviews guidelines, literature searches were conducted in PubMed, CINAHL, Ichushi-Web, CiNii, and J-Stage, without restrictions on study design or publication year. Eligible studies described camp activities for children (0-18 years) with developmental disorders in natural environments and were published in English or Japanese. Methodological quality was appraised using the Mixed Methods Appraisal Tool. Data extraction covered study characteristics, participant profiles, and reported outcomes, which were synthesized narratively.

**Results:**

Eighteen studies (15 Japanese, 3 English) met the criteria. Reported benefits included reduced anxiety, enhanced self-confidence, self-efficacy, social skills, and an increased sense of belonging. Parents experienced respite and improved family functioning. Staff and volunteers reported personal growth and a deeper understanding of disability. Natural environments were described as providing sensory stimulation, adventurous experiences, and temporary release from daily constraints, aligning with the social model of disability.

**Conclusions:**

This scoping review revealed that camp activities in natural settings hold promise as a unique integrative approach that bridges social and ecological perspectives.

## Introduction

In recent years, the increasing number of children diagnosed with developmental disorders in Japan and abroad has become a social issue ^(1), (2), (3), (4), (5)^. Developmental disorders include autism spectrum disorder (ASD), attention deficit hyperactivity disorder (ADHD), learning disabilities (LD), and others. Children with developmental disorders often face challenges with social skills and interpersonal communication, experiencing difficulties in both school and home life. Therefore, the need for social support for children with developmental disorders has been growing.

Social support includes special needs education, rehabilitation programs, social participation support, family support, and community-based programs such as camp activities. Among these, camp activities for children with developmental disorders are regarded as a multifaceted approach and have been implemented by many organizations in Japan and abroad ^(6), (7)^. Camp activities encompass a wide range of objectives spanning physical, psychological, and socio-environmental domains. From a physical perspective, they are often designed with therapeutic or rehabilitative intentions. Psychologically, they aim to foster the improvement of various skills and to provide participants with a sense of belonging. Furthermore, from a social standpoint, these activities are intended to offer peer support and respite care ^(8), (9), (10)^.

Although the effectiveness of camp activities has been mentioned in previous studies ^(11), (12)^ within the broader context of Nature-Based Interventions, these studies encompassed a wide range of activities, including horticultural therapy and animal-assisted therapy, in addition to camp activities. Therefore, they did not specifically aim to elucidate the unique value of camp activities themselves. Consequently, few studies have specifically examined the unique value and potential benefits of camp activities, which integrate educational, social, and environmental elements.

Furthermore, the social and conceptual frameworks surrounding disability and the human-nature relationship have undergone notable shifts in recent years. The traditional model, which views disability as arising from an individual’s condition or limitations, is increasingly being replaced by the “social model of disability ^(13)^,” which conceptualizes disability as a result of interactions with social and environmental factors. In addition, in the context of the relationship between nature, human health, and medicine, the concept of “planetary health” has been proposed, emphasizing the interdependence between human well-being and the health of the planet ^(14)^. Along with the growing awareness of environmental issues, this has prompted a reconsideration of the relationship between humans and nature itself.

In light of this evolving context, this study focuses on camp activities for children with developmental disorders, particularly those conducted in natural environments. The purpose of this study is to organize and clarify the value and effects of these activities, based on contemporary perspectives that reflect changing views on disability and human-nature relationships. The analysis was conducted in the form of a scoping review with a narrative synthesis.

## Materials and Methods

### Study design

This review was conducted in accordance with the Preferred Reporting Items for Systematic reviews and Meta-Analyses extension for Scoping Reviews (PRISMA-ScR).

### Search strategy

We searched for relevant articles on camp activities for children with developmental disorders in PubMed, CINAHL, Ichushi-Web, CiNii, and J-Stage, which are widely used in the medical and health sciences. The selection of these databases was also influenced by institutional access constraints. Searches were performed on May 6, 2025. No filters used for study design or publication date. The full search strategies for each database are provided in the Supplementary Material. Two authors (SN and TI) performed additional manual searches of the references in the included articles. We used the online systematic review software program Rayyan to organize article information.

### Selection process

After deleting duplicate records, we employed a two-stage study selection process. Titles and abstracts were screened for potential relevance, followed by full-text screening. Both screenings were performed independently by two authors (SN and TI). Conflicts were resolved through discussions. We used the following eligibility criteria for the selection process: (1) the content of activities for children with developmental disorders (ages 0-18, consistent with the definition of “children” in the United Nations Convention on the Rights of the Child ^(15)^ and commonly used in pediatric disability research) in natural environments was reported; (2) the article was published as an original research paper or a practical report, including gray literature such as university bulletins, with no restrictions on study design; and (3) the full text was in English or Japanese.

In accordance with prior literature on nature-based interventions ^(11), (12)^, natural environments were defined broadly to include forests, lakesides and riversides, mountainous areas, grasslands, coastal areas, and semi-natural settings such as managed campgrounds and parks that retain ecological characteristics. Borderline cases such as urban parks were screened based on the degree of artificial structures, the presence of ecological elements (e.g., vegetation, water features), and whether the primary purpose of the activities involved structured nature engagement.

**Figure 1.**
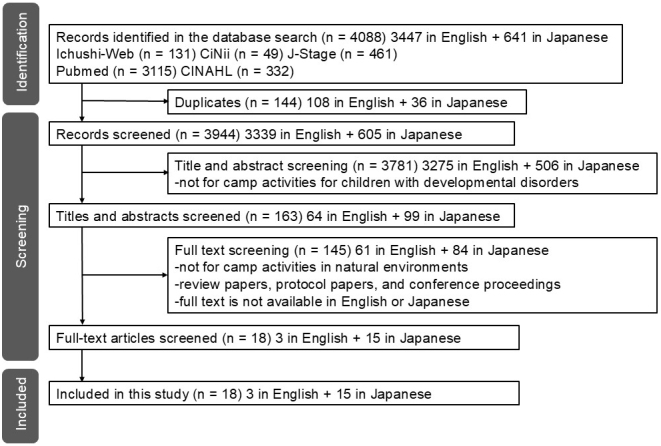
PRISMA flowchart of study selection process. The flowchart shows the number of records identified (n = 4,088), screened (n = 3,944), full-text assessed (n = 163), excluded (n = 145), and finally included in this review (n = 18). PRISMA: Preferred Reporting Items for Systematic reviews and Meta-Analyses.

At the title and abstract screening stage, studies not related to camp activities for children with developmental disorders were excluded. At the full-text screening stage, we excluded studies not set in natural environments, review articles, protocol papers, and conference proceedings, as these publication types do not provide extractable outcome data necessary for synthesis, as well as studies without full text in English or Japanese due to feasibility considerations (Figure 1).

### Quality assessment

A methodological quality assessment was conducted for all included studies using the Mixed Methods Appraisal Tool (MMAT; 2018 version) ^(16)^, which is designed to appraise the quality of qualitative, quantitative, and mixed-methods research. Two reviewers (SN and TI) independently assessed each study according to the five criteria specified for its study design category. The full wording of the five MMAT criteria (Q1-Q5) is provided in the Supplementary Material.

### Data extraction

Standardized data collection was independently performed by three authors (SN, TI, and HH) according to the PRISMA-ScR. Before data collection, the authors thoroughly discussed, tested, and revised the data collection form. The following information was obtained from each study: author name, year of publication, country of study, study design, research objectives, duration of the camp, participant types and numbers, age and sex of participating children, type of disorders, study methods, and timing of measurement.

### Synthesis of results

In addition to the above basic information, detailed descriptive data regarding the value and effectiveness of camp activities were extracted from each included study. A narrative synthesis was conducted following the guidance by Popay et al. ^(17)^, which is appropriate for scoping reviews including heterogeneous study designs and diverse outcome measures. The extracted data were tabulated, compared across studies, and inductively grouped based on themes that repeatedly appeared across the studies.

Reported outcomes were then organized according to the bio-psycho-social (BPS) model ^(18)^, categorizing findings into three domains (physical, psychological, and social). The BPS framework was adopted to capture the multidimensional nature of outcomes associated with camp programs for children with developmental disorders and because it is widely used in disability and nature-based intervention research. Two authors (SN and TI) independently extracted the data to confirm consensus.

## Results

### Overview

Eighteen references were included in the analysis (Table 1). The analysis included 15 Japanese-language references and 3 English-language references. Fifteen studies were conducted in Japan, two in the United States, and one in Canada. All 18 included studies were conducted primarily in forests, mountain campgrounds, or coastal areas, and none utilized urban parks as the primary intervention setting. The year of publication was 2001 for the oldest study, 2000-2010 for one study, 2011-2020 for nine studies, and 2021 or later for eight studies. Five were case reports, and 13 were research articles (four quantitative, seven qualitative, and two mixed-methods studies). In terms of research objectives, 15 studies focused on children with developmental disorders, two studies focused on families of children with developmental disorders, and one study focused on volunteers and staff. The duration of the camps varied: four were one-day camps, nine lasted two to three days, one lasted five days, three lasted 13 days, and one lasted 18 days. In addition, seven studies conducted a one- to three-day pre-camp prior to the main camp. The participant demographics were as follows: children in 11 studies, parents in nine studies, staff in four studies, siblings in one study, and peers in one study (with duplicates). Regarding the age ranges of children with developmental disorders, five studies focused on children aged six to 12 years, one study on those aged 13 to 18 years, one on children aged three to 12 years, four on those aged six to 18 years, and three on a broader range of three to 18 years. With regard to children with developmental disorders who attended the camp, 12 studies specified disorders, including 10 with ASD, five with ADHD, one with LD, and one with visuospatial dysfunction (with duplicates). Regarding the timing of measurement, seven conducted assessments prior to the camp (pre-camp), five during the camp, 12 immediately after the camp (post-camp), and six during a follow-up period. The longest follow-up was conducted 10 months after the camp.

**Table 1.** Overview of the 18 Reviewed Studies.

First author (country of study, year)	Study design	Research objectives	Duration	Participant demographics and sample size	Age and sex of participating children	Type of disorders	Study methods	Timing of measurement
**Thompson-Hodgetts et al. (Canada, 2024) ** ^(28)^	Qualitative & quantitative research	To explore the benefits of a brief autism education intervention on peer engagement and inclusion at day camps.	Five-day camp	Children (n = 15; intervention group = 10, control group= 5) Children with ASD (n = 5; intervention group), peers (n = 34), staff (n = 18)	Boys = 14, girls = 1; aged 4-12 Not specified	ASD	The playground observation of peer engagement, the reflexive qualitative thematic analysis approach	During camp
**Watanabe (Japan, 2024) ** ^(20)^	Qualitative research	To clarify the effectiveness and necessity of nature experience activities, as well as the difficulty of doing them alone by families.	1-day camp × 2 sessions	Parents (n = 14)	Aged 4-17	Not specified	Open-ended questionnaire, qualitative analysis based on co-occurrence network mapping	Post-camp
**Watanabe (Japan, 2023) ** ^(32)^	Qualitative research	To compare the results of a questionnaire that was administered to volunteers with no or little experience, volunteers with experience, and staff members at a children’s care center.	1-day camp × 2 sessions	University students (n = 13), volunteer staff (n = 19)	Not specified	Not specified	Open-ended questionnaire, qualitative analysis based on co-occurrence network mapping	Post-camp
**Watanabe (Japan, 2022) ** ^(19)^	Qualitative research	To investigate the effects of a nature trip to the ocean on children’s growth and development from the perspective of parents.	1-day camp × 3 sessions	Parents (n = 24)	Aged 5-15	Not specified	Open-ended questionnaire, thematic analysis	Post-camp
**Watanabe (Japan, 2022) ** ^(33)^	Qualitative research	To investigate the current situation and issues pertaining to the nature experiences from the perspective of volunteers and child day care center staff who support.	1-day camp × 3 sessions	Volunteers and staff (n = 32)	Not specified	Not specified	Open-ended questionnaire, thematic analysis	Post-camp
**Sakamoto et al. (Japan, 2022) ** ^(24)^	Quantitative research	To determine the effects of an 18-day outward-bound type camping therapy intervention on ego development and the self-concept in children with developmental disorders.	1-day orientation, 18-day main camp, 2-day follow-up	Children (n = 23)	Boys = 21, girls = 2; mean age = 13.43 ± 0.84	ASD, ADHD	Kajita’s Self-Actualization Scale, the Landscape Montage Technique	Pre-camp, post-camp, 1 month after
**Sakamoto et al. (Japan, 2022) ** ^(25)^	Quantitative research	To examine its effect on perceived self-positiveness, sense of acceptance and social skills in children with developmental disorders.	2-day pre-camp, 13-day main camp	Children with developmental disorders (n = 20); Typical development (n = 66)	Children with developmental disorders (boys = 16, girls = 4; mean age = 13.43 ± 0.84); Typical development (boys = 43, girls = 23; mean age = 12.0 ± 6.73)	Not specified	Sense of Self-Positiveness Scale (SSPS), Sense of Acceptance Scale (SAS), and Social Skills Scale in Camping (SSS-C)	Pre-camp, post-camp, 1 month after
**Otomo et al. (Japan, 2022) ** ^(31)^	Qualitative research	To clarify the factors associated with consensus-building among children with developmental disabilities in a long-term adventure camp.	2-day pre-camp, 13-day main camp	Children (n = 4)	Boys, aged 11	ASD, ADHD	participant observation, coding, and categorization	During camp
**Wenzel et al. (USA, 2020) ** ^(29)^	Quantitative research	To examine the immediate and long-term effects of participation in a family camp designed specially for families with a child with ASD.	3-day camp	Parents (n = 24) and siblings (n = 8)	Boys = 9, girls = 7	ASD	Family Leisure Activity Profile (FLAP), the Family Leisure Satisfaction Scale (FLSS), the Family Adaptability and Cohesion Scale IV (FACES IV), the Satisfaction with Family Life (SWFL), the Adaptive Skills/Level of Support Scale, relevant sociodemographic information	Pre-camp, post-camp, 2 months after, 6 months after
**Higuchi et al. (Japan, 2019) ** ^(30)^	Quantitative research	To clarify the differences in evaluations and perceptions of children’s difficulties and prosocial behavior among children, their parents, and observers.	2-day camp, 3-day camp, 2-day camp	Children (n = 15) and parents (n = 15); observers (n = 8)	Aged 9-12	ASD, ADHD	The Strengths and Difficulties Questionnaire	Pre-camp, post-camp
**Okuta and Ono (Japan, 2019) ** ^(34)^	Case report	To investigate the effectiveness of a camp for improving interpersonal relationships.	3-day preparation, 2-day camp	Parents (n = 19) and staff (n = 36)	Not specified	ASD	Open-ended questionnaire	Post-camp
**Takeuchi (Japan, 2018) ** ^(22)^	Case report	To explore how the experience affected the internal experience of a boy with developmental disabilities and to elucidate the internal processes that emerged.	1-day pre-camp, 2-day camp×3sessions	Child (n = 1) and parents	Aged 6	Visuospatial dysfunction, ADHD, LD	Interview, Projective drawing methods (pictorial diary, Baum test), questionnaire to parents, participant observation	Post-camp
**Otomo et al. (Japan, 2017) ** ^(27)^	Qualitative & quantitative research	To clarify the effects of long-term camping on its participants’ over-adaptation and the process of changing social adjustment.	2-day pre-camp, 13-day main camp	Children (n = 16; experimental group = 4, control group = 12) Child (n = 1) and parent (n = 1)	Boys = 12, girls = 4; aged 11-14 Boy, aged 11	Not specified/ADHD	Over-adaptation scale and Ibasho scale, participant observation, interview	Pre-camp, post-camp, 1 month after; during camp, post-camp
**Higuci et al. (Japan, 2017) ** ^(26)^	Quantitative research	To evaluate the effectiveness of the resilience camp.	2-day camp, 3-day camp, 2-day camp	Children (n = 15), parents and observers (n = 8)	Aged 9-12	ASD, ADHD	The Strengths and Difficulties Questionnaire	Pre-camp, post-camp
**Townsend et al. (USA, 2017) ** ^(35)^	Qualitative research	To examine the immediate and long-term influence on family well-being of participation in a family camp.	2-day camp	Parents (n = 24)	Not specified	ASD	Semi-structured interview, field notes	2 months after camp, 6 months after camp
**Onohara et al. (Japan, 2014) ** ^(23)^	Case report	To report on the implementation of the resilience camp.	2-day camp	Children (n = 25)	Boys = 9, girls = 16; aged 5-14	Not specified	Observation	During camp
**Narita et al. (Japan, 2013) ** ^(21)^	Case report	To clarify the impact of participation in camp activities on their S-HTP drawings.	1-day orientation, 3-day main camp, 1-day follow-up	Children (n = 2)	Boys; age = 15, 16	ASD	S-HTP Method	Pre-camp, 1 month after camp, 10 months after camp
**Kiya (Japan, 2001) ** ^(36)^	Case report	To examine the philosophy and content of autism camp activities, and clarify their significance and future necessity.	3-day camp	Children (n = 2)	Boys; age = 6, 15	ASD	Observation	During camp

This table summarizes study characteristics, including author (country, year), design, objectives, duration, participants, age/sex, disorder type, methods, and timing of measurement. ADHD: attention deficit hyperactivity disorder; ASD: autism spectrum disorder; LD: learning disability; N/A: not applicable.

### Quality assessment

The methodological quality assessment of the included studies, based on the MMAT (2018), revealed variability across designs (Table 2). All qualitative studies (n = 7) met all MMAT criteria, indicating methodological coherence. In contrast, non-randomized quantitative studies (n = 4) commonly showed insufficient control for confounders. Quantitative descriptive studies (n = 5) frequently demonstrated limitations in sampling strategies, measurement appropriateness, nonresponse bias, and statistical analysis. Of the mixed-methods studies (n = 2), one fulfilled all criteria, whereas the other did not adequately address inconsistencies between qualitative and quantitative findings.

**Table 2.** Methodological Quality Assessment Using MMAT (2018 Version).

First author (year)	Study design	Q1	Q2	Q3	Q4	Q5
Thompson-Hodgetts et al. (2024) ^(28)^	MM	Yes	Yes	Yes	Yes	Yes
Watanabe (2024) ^(20)^	QUAL	Yes	Yes	Yes	Yes	Yes
Watanabe (2023) ^(32)^	QUAL	Yes	Yes	Yes	Yes	Yes
Watanabe (2022) ^(19)^	QUAL	Yes	Yes	Yes	Yes	Yes
Watanabe (2022) ^(33)^	QUAL	Yes	Yes	Yes	Yes	Yes
Sakamoto et al. (2022) ^(24)^	QUAN-NR	Can’t tell	Yes	Yes	No	Yes
Sakamoto et al. (2022) ^(25)^	QUAN-NR	Can’t tell	Yes	Yes	No	Yes
Otomo et al. (2022) ^(31)^	QUAL	Yes	Yes	Yes	Yes	Yes
Wenzel et al. (2020) ^(29)^	QUAN-NR	Yes	Yes	Yes	No	Yes
Higuchi et al. (2019) ^(30)^	QUAN-NR	Yes	Yes	Yes	No	Yes
Okuta and Ono (2019) ^(34)^	QUAN-DES	Yes	No	Can’t tell	No	Can’t tell
Takeuchi et al. (2018) ^(22)^	QUAN-DES	Can’t tell	No	Can’t tell	No	Can’t tell
Otomo et al. (2017) ^(27)^	MM	Can’t tell	Yes	Yes	No	Can’t tell
Higuci et al. (2017) ^(26)^	QUAN-DES	Can’t tell	No	Yes	No	Yes
Townsend et al. (2017) ^(35)^	QUAL	Yes	Yes	Yes	Yes	Yes
Onohara et al. (2014) ^(23)^	QUAN-DES	Can’t tell	No	No	No	Can’t tell
Narita et al. (2013) ^(21)^	QUAL	Yes	Yes	Yes	Yes	Yes
Kiya (2001) ^(36)^	QUAN-DES	Can’t tell	No	Can’t tell	No	Can’t tell

This table summarizes the methodological quality assessment of the included studies, presenting study design classifications and ratings for the five MMAT criteria (Q1-Q5). Assessment outcomes are indicated as Yes, No, or Can’t tell. The full wording of each MMAT criterion (Q1-Q5) is provided in the Supplementary Material. ‘Can’t tell’ ratings primarily reflect insufficient reporting of methodological details in the original publications rather than fundamental design flaws. MMAT, Mixed Methods Appraisal Tool MM: mixed-methods; Q1-Q5: MMAT methodological quality criteria; QUAL: qualitative; QUAN-DES: quantitative descriptive; QUAN-NR: non-randomized quantitative.

### The reported aspects of camp activities for children with developmental disorders and those involved

Research on camp activities for children with developmental disorders has explored a variety of reported outcomes across different domains, including physical, psychological, and social domains.

In terms of physical experiences, camps have been reported to offer opportunities for sensory stimulation ^(19)^ and engagement in physical activities, potentially enhancing physical fitness ^(19), (20)^.

Various studies have documented changes in psychological domains, including reported reductions in anxiety ^(21)^ and potential increases in confidence ^(20), (22)^, and self-efficacy ^(23)^. Some reports indicated that camps may also contribute to the development of positive attitudes toward self-actualization ^(24)^, self-esteem ^(25)^, and self-respect ^(26)^, leading to overall mental growth ^(19)^. In the realm of social participation, there are observations of stronger feelings of acceptance ^(25)^ and a stronger sense of belonging ^(27)^ among participants. It has also been reported that conducting a brief autism education intervention during the camp can promote inclusion among participants ^(28)^. Camps are frequently aimed at the improvement of various skills. Reports suggest that camp participation may enhance social skills ^(19), (29), (30)^, social adaptability ^(27)^, consensus-building ability ^(31)^, resilience ^(23), (26), (30)^, and independent living skills ^(22)^. From the perspective of leisure support, camps have been described as providing opportunities for relaxation ^(19), (20)^, which both parents ^(19), (20)^ and staff ^(32), (33)^ recognize as a source of enjoyment for the children. Camps are also noted for their potential role in addressing experience inequality between children with and without developmental disorders ^(19), (33)^.

Of the 18 studies reviewed, 6 studies have explored the sustainability of psychological and skill-related changes associated with camp participation. One study noted that increases in attitudes and motivation toward self-actualization appeared to diminish approximately one month after the camp ^(24)^, whereas a sense of belonging was reported to remain relatively stable during the same period ^(27)^. Another study documented that self-esteem, feelings of acceptance, and social skills improved after some time had passed following participation ^(25)^. With respect to family life satisfaction, a significant increase was observed immediately after the camp, followed by a slight decline at 2 months and a subsequent increase at 6 months, remaining higher than the pre-camp level ^(29)^. Repeated participation was associated with more pronounced changes in some cases ^(20)^, and both short-term and long-term impacts were qualitatively described based on open-ended questionnaire responses, although no specific time frames were explicitly reported ^(19)^. However, the timing of the effects varied for each individual ^(32)^. It has been suggested that, as a condition for sustaining the positive changes in self-acceptance and self-concept, providing opportunities to experience success in daily life after camp participation may be important ^(24)^.

From a social perspective, effects on parents, family members, and surrounding caregivers have been noted. Four studies have explored the experiences of parents of children with developmental disorders in the context of camp activities. These studies described perceived benefits such as a sense of relief and healing ^(22), (34)^. Parents also reported reduced feelings of guilt toward siblings, a renewed understanding of family dynamics, and an increased understanding of their spouses ^(34)^. Improvements in family functioning, communication, and satisfaction with family roles and overall family life were also mentioned, with some effects reported to last up to 6 months ^(29)^. Additionally, parent-focused educational sessions conducted during the camps were described as supportive in certain cases ^(29), (35)^. Three studies have examined the perspectives of camp volunteers and staff. These reports indicated that participation in camp activities was associated with increased awareness and understanding of children with developmental disorders ^(22), (33)^ and their parents ^(22)^. In some cases, participation was also described as providing opportunities to reflect on support methods ^(22)^. Additionally, volunteers and staff were reported to enjoy the experience themselves ^(32), (33)^ and feel joy in contributing to opportunities for the children’s growth ^(32)^.

In addition, several limitations of camp activities were reported. The effects observed after participation were often described as temporary, serving primarily as a trigger for behavioral change rather than ensuring long-term sustainability ^(24)^. Participation opportunities were noted to be limited ^(34)^, and the studies that evaluated their impact generally involved small sample sizes, raising concerns regarding the generalizability of findings ^(25), (29), (31), (35)^. Furthermore, because individualized approaches were frequently required, structuring programs to accommodate diverse needs was reported as a challenge ^(36)^. From an operational standpoint, difficulties were also highlighted in relation to the sustainability of staffing and financial resources ^(20), (33), (34)^.

## Discussion

In this literature review, it was found that camps conducted in natural environments are designed with multifaceted goals, including the physical and psychological development of children with developmental disorders, their social participation, and the improvement of various skills.

When interpreting the findings of this review, it is important to consider the geographic and sociocultural context in which the majority of the included studies were conducted, as 15 of the 18 studies were carried out in Japan. Japan has historically maintained cultural, social, geographical, and policy-based traditions that emphasize coexistence with nature―for example, Shinto beliefs, which emphasize the spiritual presence of nature, and satoyama landscapes, referring to traditional rural areas characterized by harmonious human-nature interaction. In addition, Japan has a high national forest coverage of approximately 70% ^(37)^ and long-standing environmental conservation initiatives ^(38)^. These characteristics may influence how natural-environment camps are designed and implemented in the Japanese context. However, the predominance of Japanese studies makes cross-regional comparisons difficult and limits the international generalizability of this review’s findings. Therefore, caution is required when applying these results to other sociocultural contexts.

An important direction for future research concerns the appropriate duration of follow-up. The follow-up period was at most 10 months ^(36)^, and one study suggested that a six-month follow-up may be insufficient to capture meaningful changes ^(32)^. Though some reports indicate that effects may strengthen over time as experiences are integrated and reinterpreted within daily life after camp participation ^(25)^, it has also been suggested that the influence of everyday life changes and natural developmental processes makes it increasingly difficult to demonstrate direct effects attributable to camp activities as the follow-up period becomes longer ^(35), (36)^. Rather than viewing these issues merely as methodological difficulties, the present findings suggest the need for future research to establish clinically meaningful time frames for evaluating the sustainability of effects according to specific types of developmental disorders and age groups. Based on developmental considerations, future research might prioritize 6-12 month follow-ups for school-age children to capture integration into daily routines, and 12-24 month follow-ups for adolescents to assess developmental trajectory changes, while acknowledging the challenges of attributing effects specifically to camp participation as follow-up periods lengthen.

In addition to the issues concerning follow-up periods, an important point warranting further discussion is the substantial heterogeneity in intervention duration across the included studies. There was considerable variation in the duration of interventions, ranging from one day to 18 days. Although previous literature has noted that many camp programs are relatively short ^(24)^, in the studies included in this review, evaluations of shorter interventions were often based on measurements taken immediately after the camp or on observations during the program, suggesting that the timing of assessments was generally aligned with the length of the intervention. However, in none of the included studies was the rationale underlying the selection of program duration and the timing of outcome assessments explicitly described, suggesting substantial room for optimization according to program objectives and target populations. In this context, future research would benefit from the use of standardized outcome measures, pre- and post-intervention assessments, and longitudinal study designs, which may enable more systematic evaluation of changes over time and strengthen methodological rigor.

Such camps in natural settings can possibly provide non-routine and adventurous contexts ^(23), (24), (26)^, which foster cooperation ^(27), (31)^, interpersonal connections ^(25)^, recognition of individual strengths ^(27)^, and the assumption of meaningful roles ^(36)^. These extracted characteristics may be explainable within the framework of the “social model of disability ^(13)^,” a concept that has gained increasing attention in recent years. From this perspective, camp activities in natural environments can function as a place where disability is not perceived as a disability and may provide an alternative evaluation framework compared with modern school settings, where adaptability to classroom norms tends to define a “good child.” However, only one of the included studies explicitly employed the social model of disability ^(27)^, and the remaining studies can be interpreted only indirectly in relation to the core components of the social model―such as environmental adjustments, the removal of social and attitudinal barriers, and the view that individual differences should not be regarded as deficits ^(13), (39), (40)^. Therefore, in this review, the social model is positioned not as a central analytical framework but rather as a hypothetical interpretive lens that should be applied with caution. Future interdisciplinary research ―bridging medicine, education, and sociology or related fields―will be warranted to develop a more comprehensive understanding.

Future research should explore how nature-based activities foster human-nature connections ^(33)^, influence individual behaviors ^(19), (41), (42)^, and enhance environmental awareness. Although “Planetary Health” has been discussed as a relevant context ^(14)^, none of the studies included in this review explicitly addressed this concept, indicating room for future investigation.

This study has additional limitations. The search strategy was restricted to five medical and health science databases. Psychology- and education-focused databases were not included, which may have resulted in the omission of relevant studies outside the medical domain. Specifically, this may have resulted in missing studies examining camp activities from developmental psychology, educational intervention, or social learning perspectives, which could provide complementary insights to the predominantly medical- and health-oriented evidence synthesized in this review.

The MMAT-based quality assessment conducted in this review revealed substantial variability in methodological rigor across the included studies. In the narrative synthesis, findings derived from studies with methodological limitations, particularly quantitative descriptive studies, were treated as exploratory. Therefore, these methodological constraints should be taken into consideration when interpreting the overall results of this review.

### Conclusion

Camp activities have the potential to offer a unique approach that integrates sociological and ecohealth perspectives. In the contemporary medical field, where the development of interdisciplinary and integrated approaches is highly desirable, camp activities represent a possible example of such a method. However, academic validation of these activities remains insufficient, and further detailed examination of their feasibility and barriers is needed.

## Article Information

### Acknowledgments

ChatGPT (GPT-4, OpenAI) was used to assist with language editing, rephrasing, and proofreading of the manuscript between May 10 and June 30, 2025. In addition, ChatGPT (GPT-5, OpenAI) was used for the same purposes during the preparation of the revised manuscript between November 14 and December 10, 2025. The authors carefully reviewed all AI-assisted content and take full responsibility for the integrity, originality, and accuracy of the final manuscript. Hayase Hakariya is supported by the JSPS Overseas Research Fellowships, outside the submitted work.

### Author Contributions

Seiichiro Nagai and Tatsuki Ikejiri contributed to the conceptualization and interpretation of results. Seiichiro Nagai contributed to the visualization and drafting of the original manuscript. Tatsuki Ikejiri contributed to the project administration. Hayase Hakariya, Tatsuki Ikejiri, and Yuki Otsuka contributed to the critical revision of the manuscript. All authors contributed to data collection and data analysis, reviewed and approved the final version of the manuscript.

### Conflicts of Interest

The author (Seiichiro Nagai) serves as a board member of the non-profit organization ‘Yumenomori Bansousha CUE,’ which began organizing family camps for children with developmental disorders in April 2025.

### IRB Approval Code and Name of the Institution

This study was conducted using only publicly available data and did not involve any interventions or individual patient records. Thus, institutional review board approval was not necessary.

## Supplement

Supplementary Material

## References

[1] Zablotsky B, Black LI, Maenner MJ, et al. Prevalence and trends of developmental disabilities among children in the United States: 2009-2017. Pediatrics. 2019;144(4):e20190811. 31558576 10.1542/peds.2019-0811 PMC7076808

[2] Rah SS, Hong SB, Yoon JY. Prevalence and incidence of developmental disorders in Korea: a nationwide population-based study. J Autism Dev Disord. 2020;50(12):4504-11. 32347466 10.1007/s10803-020-04444-0

[3] Kuo HT, Muo CH, Chang YT, et al. Change in prevalence status for children with developmental delay in Taiwan: a nationwide population-based retrospective study. Neuropsychiatr Dis Treat. 2015;11(11):1541-7. 26203248 10.2147/NDT.S84088 PMC4487160

[4] Sasayama D, Kuge R, Toibana Y, et al. Trends in diagnosed attention-deficit/hyperactivity disorder among children, adolescents, and adults in Japan from April 2010 to March 2020. JAMA Netw Open. 2022;5(9):e2234179. 36178689 10.1001/jamanetworkopen.2022.34179 PMC9526082

[5] Sasayama D, Kuge R, Toibana Y, et al. Trends in autism spectrum disorder diagnoses in Japan, 2009 to 2019. JAMA Netw Open. 2021;4(5):e219234. 33944926 10.1001/jamanetworkopen.2021.9234 PMC8097493

[6] Takeuchi Y, Ishida Y, Noguchi K, et al. Camping for everyone : assessment of benefits for children with developmental disorders and challenges facing camping organizations. St Andrews Univ Bull Res Inst. 2020;46(1):19-36. Japanese.

[7] Clark MK, Nwokah EE. Play and learning in summer camps for children with special needs. Am J Play. 2010;3(2):238-61.

[8] Ibrahim A, Cronin KA. The impact of summer camp on social skills for children with autism spectrum disorder. J Educ Pract. 2020;11(17):129-37.

[9] Neprily K, Climie E. Happy campers: enhancing social competence in adolescents with attention-deficit/hyperactivity disorder at summer camp. J Outdoor Recreat Educ Leadersh. 2023;15(2):1-15.

[10] Wallace L. The impact of family autism camp on families and individuals with ASD. Qual Rep. 2016;21(8):1441-53.

[11] Fan MSN, Li WHC, Ho LLK, et al. Nature-based interventions for autistic children: a systematic review and meta-analysis. JAMA Netw Open. 2023;6(12):e2346715. 38060224 10.1001/jamanetworkopen.2023.46715 PMC10704280

[12] Dennis M, Henderson-Wilson C, Watson J, et al. Nature-based interventions for adults with developmental disabilities: A scoping review centering autistic adults. Sustainability. 2024;16(3):1077.

[13] Oliver M. The social model of disability: thirty years on. Disabil Soc. 2013;28(7):1024-6.

[14] Whitmee S, Haines A, Beyrer C, et al. Safeguarding human health in the Anthropocene epoch: report of the Rockefeller Foundation-Lancet Commission on planetary health. Lancet. 2015;386(10007):1973-2028. 26188744 10.1016/S0140-6736(15)60901-1

[15] Convention on the Rights of the Child [Internet]. U.N. General Assembly. 1989 [cited 2021 Dec 2]. Available from https://web.archive.org/web/20101031104336/http://www.hakani.org/en/convention/Convention_Rights_Child.pdf

[16] Hong QN, Fàbregues S, Bartlett G, et al. The Mixed Methods Appraisal Tool (MMAT) version 2018 for information professionals and researchers. Educ Inf. 2018;34(4):285-91.

[17] Popay J, Roberts H, Sowden A, et al. Guidance on the conduct of narrative synthesis in systematic reviews: A product from the ESRC methods programme. Lancaster: Lancaster University; 2006 [cited 2025 Dec 2]. Available from https://doi.org/10.13140/2.1.1018.4643

[18] Engel GL. The need for a new medical model: a challenge for biomedicine. Science. 1977;196(4286):129-36. 847460 10.1126/science.847460

[19] Watanabe K. Growth and development of mentally and developmentally disable children’s nature experiences from the perspective of parents. Stud Humanit. 2022;206:29-42. Japanese.

[20] Watanabe K. Parents’ realization and recognition of the growth of children with developmental and mental disabilities through nature activities. Bull Inst Humanit Resarch. 2023;71:59-70. Japanese.

[21] Narita N, Narita M, Tazoe M. Clinical assessment using repetitive synthetic House-Tree-Person test in autism spectrum disorder. J Jpn Soc Psychosom Pediatr. 2013;22(3):175-82. Japanese.

[22] Takeuchi Y, Sakamoto A. A case study on the positive effects of camping on a child with a developmental disorder, the Child’s family and the Camp’s staff. Japan Outdoor. Educ J. 2018;22(1):37-49. Japanese.

[23] Onohara A, Imatome T, Ishida H. The practice of resilience camps for children with developmental disorders. Bull Kansai Univ Psychol Serv Couns Cent. 2014;5:55-61. Japanese.

[24] Sakamoto A, Otomo A, Sato F, et al. Effects of long-term camping therapy on ego development and the self-concept in children with developmental disorders. Japan Outdoor. Educ J. 2022;25:1-17. Japanese.

[25] Sakamoto A, Otomo A, Maekawa M, et al. The effects of integrated long-term camp therapy on perceived self-positiveness in children with developmental disorders, and of a causality model to examine the relationship between sense of acceptance and social skills: comparison with typically developing children. Japan J Phys Educ. Hlth. Sport Sci. 2022;67:361-77. Japanese.

[26] Higuci T, Yuasa R, Ishida H. The effectiveness of the resilience camp for children with developmental disorders: evaluation using the Strengths and Difficulties Questionnaire. J Jpn Soc Psychosom Pediatr. 2017;26(3):280-5. Japanese.

[27] Otomo A, Sakamoto A. Effect of long-term camping on the social adjustment of adolescents who have psychological problem. Japan Outdoor. Educ J. 2017;21(1):29-44. Japanese.

[28] Thompson-Hodgetts S, McKillop A, et al. Influence of a brief autism education intervention on peer engagement and inclusion at mainstream day camps: a mixed-methods pilot study. J Autism Dev Disord. 2024;54(8):2860-73. 37314666 10.1007/s10803-023-06024-4

[29] Wenzel K, Townsend J, Hawkins BL, et al. Changes in family leisure functioning following a family camp for children with autism spectrum disorder (ASD). Ther Recreat J. 2020;54(1):17-31.

[30] Higuchi T, Yuasa R, Ishida H. Assessment of resilience in children with developmental disorders by the children themselves, their parents, and observers. Bull Kansai Univ Psychol Serv Couns Cent. 2019;10:21-5. Japanese.

[31] Otomo A, Sakamoto A, Sato F, et al. Consensus building among children with developmental disabilities or such tendencies who participated in a long-term adventure camp: a case study. Japan Outdoor. Educ J. 2022;25:73-90. Japanese.

[32] Watanabe K. Significance of co-education for university students to learn together with children with intellectual and developmental disabilities through support for nature experience activities. Stud Humanit. 2023;210:35-49. Japanese.

[33] Watanabe K. Significance and issues of nature experiences of children with developmental disorders and mental disabilities. Stud Humanit. 2022;205:59-74. Japanese.

[34] Okuta N, Ono A. Intervention programs to improve health exercise, diet and Interpersonal relations of children with developmental disabilities. Health Psychol Res. 2019;31(Special issue):245-52. Japanese.

[35] Townsend JA, Van Puymbroeck M. Parental perceptions of changes in family well-being following participation in a camp experiences of families with a child with ASD. Ther Recreat J. 2017;51(2):143-63.

[36] Kiya H. A discussion of therapeutic treatment and support system for children and adults with autism : from the practice of ‘Wakasugi camp for autism.’ Bull Integr Cent Educ Res Train. 2001;12:19-28. Japanese.

[37] Forest coverage rate and plantation forest rate by prefecture [Internet]. Forestry Agency. 2022 [cited 2021 Dec 8]. Japanese. Available from: https://www.rinya.maff.go.jp/j/keikaku/genkyou/r4/1.html

[38] Takeuchi K. Rebuilding the relationship between people and nature: the Satoyama Initiative. Ecol Res. 2010;25(5):891-7.

[39] Shakespeare T. Disability rights and wrongs. London: Routledge; 2006. 240 p.

[40] World Health Organization. International classification of functioning, disability and health (ICF). Geneva: World Health Organization; 2022 [cited 2025 Dec 2]. Available from: https://iris.who.int/items/ffa4b5b5-378c-46a1-acdc-7ef20506a3f8

[41] Zafeiroudi A. Enhancing adolescents’ environmental responsibility through outdoor recreation activities. Acad J Interdiscip Stud. 2020;9(6):43.

[42] San Jose AL, Nelson KE. Increasing children’s positive connection to, orientation toward, and knowledge of nature through nature camp experiences. Int J Environ Sci Educ. 2017;12(5):933-44.

