# Supplementary material for: A Scoping Review of Camp Activities for Children with Developmental Disorders
Source: JMA J. 2026 Mar 19;9(3):583–91. doi: 10.31662/jmaj.2025-0435 (PMC13246261; doi:10.31662/jmaj.2025-0435)
Supplement: Supplementary Material [file 2433-3298-9-3-0583-s001.pdf]

Supplementary Material

Search Strategies

For English-language literature, PubMed and CINAHL were used, with (“camp*” OR “outdoor experience*” OR “outdoor activity*” OR “nature experience*”) AND (“developmental disorder*” OR “attention deficit hyperactivity disorder*” OR “autism spectrum disorder*” OR “learning disability*”). For Japanese literature, Ichushi-Web, CiNii, and J-Stage were used, with (“キャンプ” OR “野外体験” OR “野外活動” OR “自然体験”) AND (“発達障害” OR ”注意欠陥多動性障害” OR “自閉スペクトラム症” OR “学習障害”).

MMAT criteria (Q1–Q5)

**Qualitative**	**Q1**	Is the qualitative approach appropriate to answer the research question?
	**Q2**	Are the qualitative data collection methods adequate to address the research question?
	**Q3**	Are the findings adequately derived from the data?
	**Q4**	Is the interpretation of results sufficiently substantiated by data?
	**Q5**	Is there coherence between qualitative data sources, collection, analysis and interpretation?
**Non-randomized quantitative**	**Q1**	Are the participants representative of the target population?
	**Q2**	Are measurements appropriate regarding both the outcome and intervention (or exposure)?
	**Q3**	Are there complete outcome data?
	**Q4**	Are the confounders accounted for in the design and analysis?
	**Q5**	During the study period, is the intervention administered (or exposure occurred) as intended?
**Quantitative descriptive**	**Q1**	Is the sampling strategy relevant to address the research question?
	**Q2**	Is the sample representative of the target population?
	**Q3**	Are the measurements appropriate?
	**Q4**	Is the risk of nonresponse bias low?
	**Q5**	Is the statistical analysis appropriate to answer the research question?
**Mixed-methods**	**Q1**	Is there an adequate rationale for using a mixed methods design to address the research question?

	**Q2**	Are the different components of the study effectively integrated to answer the research question?
	**Q3**	Are the outputs of the integration of qualitative and quantitative components adequately interpreted?
	**Q4**	Are divergences and inconsistencies between quantitative and qualitative results adequately addressed?
	**Q5**	Do the different components of the study adhere to the quality criteria of each tradition of the methods involved?
